# Diabetic Polyneuropathy in Type 2 Diabetes Mellitus: Inflammation, Oxidative Stress, and Mitochondrial Function

**DOI:** 10.1155/2016/3425617

**Published:** 2016-12-12

**Authors:** Luis Miguel Román-Pintos, Geannyne Villegas-Rivera, Adolfo Daniel Rodríguez-Carrizalez, Alejandra Guillermina Miranda-Díaz, Ernesto Germán Cardona-Muñoz

**Affiliations:** ^1^Departamento de Ciencias de la Salud-Enfermedad, Centro Universitario de Tonalá, Universidad de Guadalajara, Guadalajara, JAL, Mexico; ^2^Unidad de Investigación Médica, Instituto de Investigación Clínica de Occidente, Guadalajara, JAL, Mexico; ^3^Instituto de Terapéutica Experimental y Clínica, Departamento de Fisiología, Centro Universitario de Ciencias de la Salud, Universidad de Guadalajara, Guadalajara, JAL, Mexico

## Abstract

Diabetic polyneuropathy (DPN) is defined as peripheral nerve dysfunction. There are three main alterations involved in the pathologic changes of DPN: inflammation, oxidative stress, and mitochondrial dysfunction. Inflammation induces activation of nuclear factor kappa B, activator protein 1, and mitogen-activated protein kinases. Oxidative stress induced by hyperglycemia is mediated by several identified pathways: polyol, hexosamine, protein kinase C, advanced glycosylation end-products, and glycolysis. In addition, mitochondrial dysfunction accounts for most of the production of reactive oxygen and nitrosative species. These free radicals cause lipid peroxidation, protein modification, and nucleic acid damage, to finally induce axonal degeneration and segmental demyelination. The prevalence of DPN ranges from 2.4% to 78.8% worldwide, depending on the diagnostic method and the population assessed (hospital-based or outpatients). Risk factors include age, male gender, duration of diabetes, uncontrolled glycaemia, height, overweight and obesity, and insulin treatment. Several diagnostic methods have been developed, and composite scores combined with nerve conduction studies are the most reliable to identify early DPN. Treatment should be directed to improve etiologic factors besides reducing symptoms; several approaches have been evaluated to reduce neuropathic impairments and improve nerve conduction, such as oral antidiabetics, statins, and antioxidants (alpha-lipoic acid, ubiquinone, and flavonoids).

## 1. Introduction

Diabetes mellitus (DM) leads to important morbidity and mortality, consequence of macro- and microvessels complications [1]. Type 2 DM is characterized by insulin resistance, with or without insulin deficiency that induces organ dysfunction [2]. Persistent hyperglycemia in DM generates reactive oxygen species (ROS) and nitrosative species (RNS); both are considered an essential factor for DM macro- and microvessels complications [3]. Along with overproduction of ROS and RNS, a reduction of the activity of antioxidant enzymes is known to cause endothelial dysfunction, insulin resistance, and DM complications [4]. Furthermore, oxidative stress inhibits insulin secretion in pancreatic *β*-cells by activation of uncoupling protein 2 (UCP-2), which, in turn, reduces the adenosine triphosphate (ATP)/adenosine diphosphate (ADP) ratio, and thus reduces the insulin-secretory response [5]. This approach explains the pancreatic dysfunction induced by glucose toxicity, as part of the pathophysiology of DM.

ROS and RNS are responsible for structural derangement of carbohydrates, proteins, lipids, and nucleic acids [6]. These free radicals activate different signaling pathways, which leads to transcriptional genes related to diabetic complications; activation of nuclear factor kappa B results in induction of proinflammatory proteins, also observed in diabetic polyneuropathy (DPN) [7]. In this review, we aimed to describe the behavior of inflammatory response, oxidative stress, and mitochondrial function in DPN of type 2 DM.

## 2. Diabetic Polyneuropathy

Diabetic neuropathies are a heterogeneous group of pathological manifestations with the potential to affect every organ, with clinical implications such as organ dysfunction, which leads to low quality life and increased morbidity [8]. DPN is defined as peripheral nerve dysfunction with positive and negative symptoms [9]. Some authors describe it as the most frequent microvessel diabetic complication, and it is present in approximately 10% of recently diagnosed diabetic patients [10, 11].

### 2.1. Epidemiology

For the analysis of prevalence of DPN we included some of the most representative studies by country. Noteworthy, we only report the prevalence of type 2 DM patients with the information contained in each article. The distinction of the population where the prevalence is reported is important due to the particular characteristics of the evaluated patients. There are several biases to take into consideration in particular with each study: intra- or interobserver variability, examiners ability to perform the tests, different detection methods, presence/absence of arteriopathy, and comorbidities, among others. Ziegler et al. minutely described the prevalence of DPN from 1986 to 2009 and separated the known information in type 1 and 2 DM [12].

**Figure 1:**
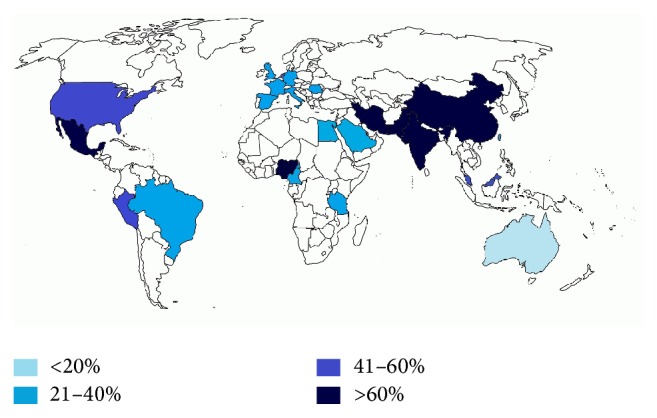
Prevalence of DPN by country. Colors represent the percentiles 25, 50, and 75 of epidemiology studies.

The prevalence of DPN ranged from 2.4 to 78.8%, both in Chinese population. The median prevalence of evaluated studies was 59%. We established percentiles around the minimum and maximum prevalence reported and divided those reported by country into the following: above percentile 75 (>60% of DPN), higher than percentile 50 (41–60%), between percentile 25 and 50 (21–40%), and below percentile 25 (<20%).  Figure 1 resumes the highest prevalence reported by country [13–44].

There was one study performed in Spain where the prevalence was reported as low as 1.33%; however, the method of detection was not reported, and apparently the patients were classified using their hospital discharge records; thus, we did not include this study in the analysis because we believe it lacks essential information [45]. In another study from Germany and UK type 2 DM patients who were recruited from general practice nationwide, the authors used the 10th revision of the International Statistical Classification of Diseases and Related Health Problems (ICD-10) to establish the prevalence of DPN in their communities, but then again they do not report the diagnostic method employed by the physicians: in Germany it was 5.7% and in the UK 2.4% [46]. One study that explores the presence of painful neuropathy demonstrated a prevalence of mild symptoms of 14%, moderate 18%, and severe 16%: the overall presence of painful neuropathy was 34% [47]. Davies et al. also reported the prevalence of painful DPN in 269 patients with type 2 DM of a population-based sample recruited in an urban community in South Wales, UK; the results were an overall prevalence of 26.4% patients with DPN: neuropathic pain 19%, nonneuropathic pain 36.8%, mixed pain 7.4%, and no pain 36.8% [18]. However, some authors report a prevalence up to 77.4% in some series [33].

### 2.2. Risk Factors

The Rochester Diabetic Neuropathy Study (RDNS) is a classical cohort in the 90s that gives us plenty of information regarding demographic and clinical outcomes in this area. The classical variables which influence neuropathic endpoints are age, gender, height, weight, body surface area, and body mass index (BMI) [48]. The temperature also influences the measures, as shown in nerve conduction studies of normal subjects, and in consequence the protocols for nerve conduction studies include having a standardized normal temperature room [49, 50].* Age* is the most evaluated risk factor in the majority of epidemiological studies, with those ≥70 years old considered the most prevalent group for DPN (odds ratio [OR] 1.073 [95% CI 1.051–1.097], *p* < 0.001); it is considered a risk factor for symptoms, deficits, and sensation perception deficits, so much for presence and severity of DPN [17, 19, 39, 42, 43]. Disparity results are found regarding* gender*, with some authors reporting male sex as a severity risk factor (OR 2.01, *p* = 0.02) [19, 51]. A more recent report found that male gender in type 2 DM patients from a survey conducted in a tertiary hospital showed an OR of 2.7 (95% CI 1.4–5.1, *p* = 0.001). However, in patients with established DPN, apparently female gender is associated with more symptomatic disease and severity of pain [42]. Furthermore, one study demonstrated that, after adjustments for age, diabetes duration, and differences in clinical neuropathy, women still had a 50% increased risk of painful symptoms compared with men (OR 1.5 [95% CI 1.4–1.6], *p* = 0.0001) [47, 52]. One study demonstrated the correlation between* height* and nerve conduction studies in normal subjects from 17 to 77 years old. A strong inverse correlation was found between height and sural (*r* = −0.7104), peroneal (*r* = −0.6842), and tibiae (*r* = −0.5044) conduction velocities and is also correlated to nerve latencies (sural *r* = 0.6518, peroneal *r* = 0.4583, tibiae *r* = 0.7217, and median *r* = 0.5440) [49].* Overweight and obesity* are considered as risk factors for the presence of DPN with OR 1.036 (95% CI 1.005–1.068, *p* = 0.022) [14]. Furthermore,* weight* by itself is also a risk factor with OR 1.01 (95% CI 1.00–1.03, *p* = 0.044) [28]. A study performed from 2010 to 2012 in 16 diabetes outpatient clinics in Japan, where 298 patients were included, reported that overweight and obesity are risk factors for pain and numbness in patients with DPN [52]. In type 2 DM patients with BMI ≥ 25, age (OR 1.016 [95% CI 1.008–1.024]), duration of DM (OR 1.072 [95% CI 1.056–1.087]), and HbA1c (OR 1.053 [95% CI 1.013–1.095]) are considered risk factors for the presence of DPN [32]. Dyck et al. established the risk factors for DPN in 264 diabetic patients of both type 1 and 2 DM and found that more than 20 covariates were statistically associated with DPN severity. They divided the risk factors for both type 1 and type 2, type 1 DM only, and type 2 DM only; after inclusion of the variables in a multivariate analysis and excluding markers of microvessel and macrovessel disease, for type 2 DM, an altered* glycated hemoglobin* (HbA1c) was the most significant independent risk factor associated with severity [53]. Moreover, a quantitative assessment of nerve conduction studies in patients with type 2 DM found that increased HbA1c is a risk factor for severity of DPN, resulting in an OR of 5.233 (95% CI 1.700–16.103), *p* = 0.004) [54]. These findings have been constantly reported, with a more recent study associating the presence of an altered HbA1c with DPN (OR 1.139 [95% CI 1.021–1.271]) [39] and >50% of patients with DPN having HbA1c ≥ 7% [19, 43]. Another risk factor is longer* diabetes duration*, with adjusted OR 1.05 (95% CI 1.02–1.08) [19, 28, 39].* Ethnicity* also influences severity of DPN, being the black non-Hispanic, mixed, or Asian patients more affected than Caucasians [19, 42]. A higher* education* level is a protective factor for DPN in type 2 DM (OR 0.72 [95% CI 0.58–0.88, *p* < 0.05]). A history of* hypertension* is more frequently found in patients with type 2 DM with DPN and coronary artery disease (CAD) in the BARI2 cohort, although no regression analysis was made [19].

Some authors have also considered pharmacological treatments for DM, such as* insulin*, as a risk factor for the presence of DPN (OR 1.57 [95% CI 1.15–2.13]), and related with greater risk of numbness (OR 3.21 [95% CI 1.52–6.97]), after adjustment for duration of diabetes, HbA1c levels, and age [19, 52]. Painful symptoms also seem to be more prevalent when patients are treated with insulin, compared with those on oral hypoglycemic agents and diet-only (54.7, 50.6, and 42.1%, respectively; *p* = 0.0001) [47].

DPN is strongly associated with diabetic retinopathy (OR 1.10, *p* < 0.01), not considered as a risk factor, but part of the same physiopathological cause for microvessel disease in DM [51, 53]. DPN is also more frequent in patients with nephropathy compared with those without overt nephropathy (62% versus 32%), although not statistically different when evaluated in 156 type 2 DM patients. Macrovessel disease is also associated with DPN; peripheral vascular disease is three times more common in patients with DPN (OR 2.31, *p* = 0.007) [51].

Modern risk factors have been explored lately, with interesting results addressing more complex determinations. Ankle-brachial index as a marker for peripheral arterial disease constitutes an independent predictor factor for neuropathy (OR 2.260 (95% CI 1.324–3.858, *p* = 0.003) [39]. In a hospital-based report in type 2 DM patients from Sub-Saharan Africa, the determinants of polyneuropathy were urban residence (OR 7.9 [95% CI 1.4–44.9], *p* = 0.02), infection with hepatitis C virus (OR 4.8 [95% CI 1.1–21.4], *p* = 0.002), infection with HIV (OR 3.4 [1.1–10.5], *p* = 0.012), and presence of albuminuria (OR 20.4 [95% CI 6.5–63.9], *p* = 0.0001) [33]. The latter was also reported previously in patients with type 2 DM and coronary artery disease as more prevalent in patients with DPN [19].

An association between cardiovascular disease and DPN has been mentioned previously in between lines; however, there were no adapted studies that addressed this particular issue until 2014, where Ybarra-Muñoz et al. demonstrated that patients with type 2 DM and cardiovascular disease presented an increased risk of developing DPN at 10 years of follow-up (OR 2.32 [95% CI 1.03–5.22], *p* = 0.04). It was performed in a primary care setting from 2002 to 2012, and the selection of patients was according to the presence of previous history of myocardial infarction, angina, coronary artery disease, bypass-grafting, stroke, peripheral vascular disease, or ischemic changes detected on a 12-lead electrocardiogram [55].

### 2.3. Pathophysiology

**Figure 2:**
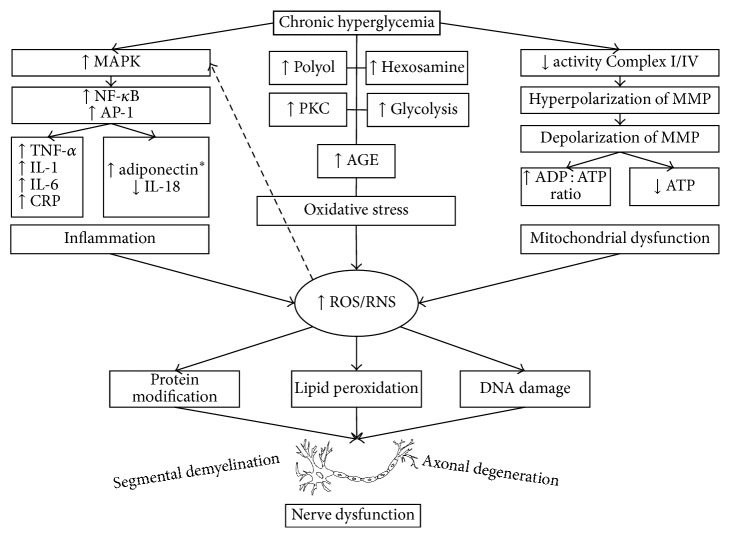
Mechanisms of nerve dysfunction induced by hyperglycemia. The description of how inflammation, oxidative stress, and mitochondrial dysfunction contributes to ROS/RNS formation and nerve damage. ^*∗*^Paradoxical increase of adiponectin in DPN.

Three main characteristics are involved in the pathologic changes of DPN, namely, inflammation, oxidative stress, and mitochondrial dysfunction (Figure 2). They account for most of the pathologic processes that affect microvessels and nerve fibers. It is now known that there is a structural abnormality of nerve capillaries in DM and an association between pathologic abnormality of vessels and pathologic abnormality of nerve fibers [56].

Biopsy findings reflect a loss of multifocal and focal proximal nerve fibers, but a more severe damage in distal fibers; the number of myelinated fibers diminishes from proximal to distal nerves [57]. Axonal degeneration and segmental demyelination are the main pathological characteristics of neuropathic damage induced by hyperglycemia. It has been established that the first pathologic changes in DPN are axonal degeneration with subsequent regeneration, but insufficient to reestablish the structural abnormalities due to chronic hyperglycemia. Onion bulbs are shown in nerve biopsies, characteristic of hypertrophic neuropathy, representing the demyelination and remyelination of the nerve fibers [57].

The number of capillaries per mm^2^ and the minimum intercapillary distance are not affected in DM patients; however, the percentage of capillaries closed is higher in subjects with DPN compared with healthy controls and also related to the severity of the neuropathy [58]. The walls of microvessels are thickened, due mainly to excessive numbers of basement membranes, and undergo degenerative changes, which cannot be explained by age. Degeneration of both endothelial and periendothelial cells is considered characteristic changes in DPN; however, some authors have shown that transformation of the microvascular wall rather than a process of microvascular de- and regeneration is the main pathological change [59]. In streptozotocin-diabetic rats, axonal outgrowth of dorsal root ganglion neurons exhibited a twofold elevation of ROS in axons after 24 hours of 25 mmol/L of glucose exposure compared to controls, predisposing the axon to degeneration and dissolution [60].

#### 2.3.1. Inflammation

The ROS-mediated inflammation induces activation of nuclear factor kappa B (NF-*κ*B), activator protein 1 (AP-1), and mitogen-activated protein kinases (MAPK). NF-*κ*B at the same time facilitates the production of inflammatory cytokines: tumoral necrosis factor alpha (TNF-*α*), interleukin- (IL-) 6, cyclooxygenase 2 (COX-2), and inducible nitric oxide synthase (iNOS). The MAPK is promoted by hyperglycemia through apoptosis signaling kinase 1 or directly by ROS and causes activation of cytosolic NF-*κ*B [61]. It is known that Nrf2 consist in the counterpart of NF-*κ*B maintaining a cellular homeostasis, but ROS overproduction can generate an imbalance between them and associates with nerve damage [62, 63].

Adipocytokines are inflammatory substances secreted by fat tissue, which includes TNF-*α*, adiponectin, and leptin [64]. In overweight and obese patients a low insulin sensitivity and adiponectin levels have been found, and they had a positive correlation. Inversely, TNF-*α* receptors had a negative correlation with insulin sensitivity, which leads to the conclusion that TNF-*α* might be associated with insulin resistance through its relationship with substrate oxidation in hyperinsulinemic condition and nonoxidative glucose metabolism [65]. Furthermore, hypoadiponectinemia is related to higher fasting plasma glucose and triglycerides, lower HDL cholesterol, and visceral obesity and also to higher levels of inflammatory cytokines (IL-6 and IL-1) and C reactive protein (CRP) in type 2 DM subjects [66].

From 2002 and 2004 a total of 105 type 2 DM subjects were evaluated to find the role of adipocytokines in DPN. Matsuda et al. found a relationship between leptin and TNF-*α* with sural conduction velocity in patients with DPN; however, the study lacked plausibility criteria for it was a cross-sectional study, and they had difficulty explaining these correlations [67]. Years later, in 2009, the clinical implications of inflammation in DPN were addressed again, with enough findings to establish the association of inflammation markers with DPN. Herder et al. studied a total of 10 inflammation biomarkers in 227 type 2 DM patients selected from a population-based survey in Germany and found that higher levels of CRP and IL-6 were most consistently associated with DPN compared to type 2 DM patients without neuropathy. These two markers were directly associated with presence and severity of DPN, while IL-18 was inversely related to neuropathy [68].

In type 2 DM patients of recent diagnosis, an increase of IL-6, total adiponectin, and high molecular weight adiponectin were found in subjects with DPN. These results seem to be counterintuitive, since adiponectin is considered an anti-inflammatory cytokine; however, it has been suggested that adiponectin could be upregulated by metabolic and/or inflammatory insults. A positive association of IL-6 and adiponectin with the presence of DPN was found, after adjusting for age, sex, time since diagnosis of diabetes, HbA1c, waist circumference, height, total cholesterol, hypertension, current smoking, physical activity, use of lipid-lowering medication, use of NSAIDs, history of myocardial infarction, and/or stroke; therefore, it can be used as an independent marker for the presence of DPN [69]. Also, in type 2 DM patients with DPN, an inverse association between TNF-*α* and nerve conduction parameters has been proven, mainly for sural, median, and ulnar nerve conduction velocity. This inflammatory marker was tested in patients without DPN or with less than 8 years and >8 years from the diagnosis of DPN. The serum levels of TNF-*α* were markedly raised in neuropathy patients and the trend continued when duration of disease increased [70]. Another study included patients aged from 61 to 82 years with DM and DPN from the population-based Cooperative Health Research in the Region of Augsburg (KORA) F4 study and found that serum concentrations of IL-1 receptor antagonist (IL-1RA) were positively associated with the presence of DPN and higher Michigan Neuropathy Screening Instrument (MNSI) scores in age-adjusted and sex-adjusted analyses, reassuring the association of inflammatory cytokines in the older population [44]. A restriction of glucotoxicity from the diet has demonstrated that inflammatory changes are diminished in diabetic patients, an improvement of insulin resistance has been observed, and a reduction of leptin and an increase of adiponectin levels have been reported [71, 72].

#### 2.3.2. Oxidative Stress

There are several pathways for ROS production, such as glycolysis, hexosamine pathway, protein kinase C (PKC) pathway, polyol pathway, and autoxidation [73]. High extracellular glucose enhances glucose activation and the* glycolytic pathway*, resulting in an enhanced pyruvate formation. Pyruvate oxidation in the mitochondria is associated with an increase in the mitochondrial membrane potential (MMP), and high MMP is responsible for an overproduction of ROS, which in turn inhibits glycolysis by a negative feedback. Hence, the flux of carbon is then rerouted towards the glucosamine pathway, which is responsible for the transcriptional consequence of high extracellular glucose [74]. Moreover, an inhibition of glucose-6-phosphate dehydrogenase leads to an increase of free radicals through the production of nicotinamide adenine dinucleotide phosphate (NADPH), induced by hyperglycemia [75]. In* hexosamine pathway*, fructose-6-phosphate is diverted from glycolysis to produce glucosamine-6-phosphate, which in turn converts into uridine diphosphate- (UDP-) N-acetyl glucosamine (UDP-GlcNAc), a substrate for the formation of proteoglycans and other glycoproteins [76]. The UDP-GlcNAc can inhibit the endothelial nitric oxide synthase (eNOS) activity and induce an increased expression of transforming growth factor beta (TGF-*β*) [77–79]. Intracellular hyperglycemia also induces the formation of diacylglycerol (DAG) from the glycolytic intermediate dihydroxyacetone phosphate, through reduction of the latter to glycerol-3-phosphate. Increased DAG activates* protein kinase C*, but the polyol pathway can also activate some PKC isoforms, particularly relevant to explain microvascular complications of DM [80]. The excess of glucose can be diverted to the* polyol pathway* by the enzyme aldose reductase to produce sorbitol, which is then accumulated in nerve fibers, and reduce the levels of myoinositol [81]. In consequence, there is a reduction in the axon capacity to propagate the membrane action potential and diminished capacity of nerve regeneration [82, 83]. In rat model, an association between exhaustion of myoinositol and a reduction of the ATPase induces a nerve conduction deficit [84].* Autoxidation* is considered a mechanism where glucose itself can be toxic as a result of a nonenzymatic glucose binding to proteins, resulting in advanced glycation end stage (AGE) products [85]. An increase of AGE in axons and Schwann cells have been reported in peripheral nerves of patients and animal model of DM [86]. It is a fact that AGE are related to the progression of type 2 DM complications, including DPN, diabetic nephropathy, myocardiopathy, peripheral artery disease, and retinopathy [87]. Experimental studies in rats showed that, after a high AGE diet during a long period of time, insulin resistance appears, and type 2 DM [88]. 

*Oxidative Stress Biomarkers.* The ROS and RNS are intended to induce apoptosis of dysfunctional cells and recycling some of their components; however, imbalance occurs when the antioxidant capacity of patients with DM is exceeded with the production of free radicals. The ROS include free radicals, such as superoxide (O^2•−^), hydroxyl (HO^•^), peroxyl (RO^2−•^), and hydroperoxyl (^•^HRO^2−^  ), and nonradical species, such as hydrogen peroxide (H_2_O_2_) and hydrochloric acid (HOCl). Among RNS we can find free radicals as nitric oxide (NO^•^) and nitrogen dioxide (NO^2•^) and nonradical peroxynitrite (ONOO^−^), nitrous oxide (HNO^2^), and alkyl peroxynitrates (RONOO) [89].

Oxidative and nitrosative stress markers have been extensively studied in DPN, and the relationship between ROS and neuropathy in DM has been assessed in vitro and in vivo. A loss of 53% of large myelinated fibers due to ROS overproduction in chronically diabetic animals has been proven: variations in basal glucose as small as 10 mM induce neuronal injury [90]. In vitro and in vivo studies have shown an increase in oxidative stress biomarkers in lipids (thiobarbituric acid-reactive substances [TBARS], malondialdehyde [MDA], and isoprostanes), proteins (protein carbonylation and nitrosylation), carbohydrates (AGE products), and DNA (8-hydroxy-deoxyguanin), along with inhibition of endogenous antioxidant synthesis [91]. Ziegler et al. found significant elevations in three reliable oxidative stress markers, plasma 8-iso-prostaglandin F_2*α*_ (8-iso-PGF_2*α*_), O^2•−^ and ONOO^−^ of patients with DM and DPN, compared to healthy controls and diabetic patients without DPN. In multivariate models, O^2•−^ and ONOO^−^ were independently associated with neuropathic deficits and related to the presence and severity of DPN [92].

Free radicals have the capacity to attach to membranes and induce cell damage; when they affect structural lipids of cells is called lipid peroxidation (LPO), and MDA is considered an index of endogenous LPO [93, 94]. A significant elevation in LPO markers like MDA and TBARS of patients with type 2 DM are a consistent finding in several publications [95–98]. Moreover, when type 2 DM patients exhibit DPN, MDA levels are ~40% higher than diabetics without neuropathy and almost three times higher than healthy controls, with similar increases in total antioxidant capacity [99]. The TBARS are also increased in patients with DPN compared to type 2 DM subjects without neuropathy [100] suggesting that oxidative stress is an important if not essential pathophysiologic process for DPN. The increase in ROS and impaired regulation of oxidative stressors result in programmed cell death of neurons and provide a mechanism to explain how impaired regulation of peak glucose levels leads to ROS-induced injury in diabetic neuropathy [90]. 

*Antioxidant Status.* Antioxidants participate in mechanisms to reduce the deleterious effects of free radicals by preventing their production and/or inactivating them through enzymatic defense systems. The most studied endogenous antioxidants are superoxide dismutase (SOD), catalase (CAT), glutathione S-transferase (GST), and glutathione peroxidase (GPx) and nonenzymatic antioxidants as uric acid, carotenoids, flavonoids, and lipoic acid vitamins A, C, and E. Aydin et al. selected type 2 DM patients from an outpatient clinic of an endocrinology department and evaluated their levels of copper zinc SOD, erythrocyte and plasma selenium dependent GPx, and erythrocyte CAT activities, before and after treatment with oral antidiabetics (OA) comparing them with healthy controls. The results were an increase in levels of SOD but normal activity of GPx and CAT. All antioxidants reduced after 3 months of treatment with OA compared to controls, probably by ameliorating the hyperglycemic state and/or the powerful O^2−^ radical scavenging activity of oral antidiabetics [97]. Furthermore, vitamin E-to-lipid ratio and vitamin C, two potent endogenous antioxidants with well-identified plasma scavenging features, are decreased in patients with type 2 DM and DPN [92]. Similar reports have been published regarding the antioxidant status of type 2 DM patients, where an increased activity is found in hyperglycemic conditions [92, 98], and higher elevations are shown in patients with DPN [100].

#### 2.3.3. Mitochondrial Dysfunction

Mitochondria are intrinsically associated with ROS production; its normal function is altered by hyperglycemia [101]. More than 90% of ROS are generated in the mitochondria [102]. The damage to this organelle can lead to cellular apoptosis and/or reduce the capacity to generate ATP, finally altering the axon through its degeneration. A lower activity of complex I and complex IV secondary to reduced protein expression of certain complex components has been shown in animal models, and an impaired electron transport chain function also causes an increase in ROS generation [103, 104]. The electron chain transport induces the production of O^2•−^ as an end-product from electron uncoupling, followed by a reduction in oxygen to form this free radical. Moreover, mitochondria can also generate HO^•^, H_2_O_2_, and NO^•^, capable of causing deleterious effects to other proteins or the DNA [62]. The nervous system seems to be particularly vulnerable to oxidative stress damage due to a high energetic demand and elevated lipids content [105]. When hyperglycemia is controlled, the mitochondria experience a reduction of O^2•−^ inhibition, along with improvement of mitochondrial function and DNA [106].

It has been proved that hyperglycemia induces a dose-dependent effect on cleavage of caspases through ATP depletion. Hyperglycemia generates ROS coupled with hyperpolarization of the mitochondrial membrane potential (MMP), followed by mitochondrial membrane depolarization, which is temporally related to an increase in ADP : ATP ratio and an absolute decrease in ATP levels. This in turn is coupled with cytochrome *c* release from the intermitochondrial membrane space and cleavage of caspases, resulting in dorsal root ganglion apoptosis [90].

In diabetic dorsal root ganglion neurons exposed to increase concentrations of glucose both in vivo and in vitro, there is a loss of electrons from the mitochondrial electron transfer chain, coupled with initial hyperpolarization of the MMP, and it results in generation of excess ROS in the mitochondria; in turn, there is increased mitochondrial injury, mitochondrial membrane depolarization, and swelling, with release of apoptosis-inducing factors from the mitochondria into the cytosol, leading to formation of an apoptosome [107]. The relation between ROS overproduction and mitochondrial dysfunction relies in these findings: high glucose increases ROS, destabilizes the MMP, and induces mitochondrial apoptosis [90, 107]. However, there is more than one pathway for ROS increase, as demonstrated by Akude et al., who analyzed the proteins associated with mitochondrial dysfunction, oxidative phosphorylation, ubiquinone biosynthesis, and the citric acid cycle and found that these were downregulated in diabetic samples. Respiration and mitochondrial complex activity was significantly decreased by 15 to 32% compared with control, which leads to reduced levels of intramitochondrial O^2•−^. Even so, the axons of diabetic neurons exhibited oxidative stress and depolarized mitochondria, concluding that alternative pathways appear to contribute to raised ROS in axons of diabetic neurons under high glucose concentration [108].

### 2.4. Diagnosis

There is no gold standard for the diagnosis of DPN. The expert panel of San Antonio conference recommends that it should be made on the basis of neuropathic symptoms, signs, and nerve conduction studies (NCS) [109]. In 2005 a report of the American Academy of Neurology, the American Association of Electrodiagnostic Medicine, and the American Academy of Physical Medicine and Rehabilitation developed a definition of distal symmetric polyneuropathy (DSPN) that served as a basis for actual research studies. It was established that electrodiagnostic studies are not required for field or epidemiologic studies; therefore, many studies lack nerve conduction studies as part of the evaluation [50]. However, a combination of symptoms and sings is required to increase the specificity of the tests, and therefore, more consideration on the results should be made while interpreting the prevalence in type 2 DM.

A classification for DPN has been proposed for clinical and research purposes by the Toronto Diabetic Neuropathy Expert Group. The following definitions are included:* possible*, when symptoms or signs are present;* probable*, diagnosed with a combination of symptoms and signs of neuropathy;* confirmed*, when NCS are abnormal and symptoms/signs are present. A fourth classification (subclinical) is appointed to those patients without clinical findings but abnormal NCS or a validated measure of small fiber neuropathy may be used. Among these, nerve biopsy, skin biopsy (morphometric quantification of intraepidermal nerve fibers), corneal confocal microscopy, and nerve axon reflex/flare response are considered validated tools. However, subclinical tests for DPN are only recommended for research studies [110].

To assess the severity of DSPN, several approaches can be recommended: the graded approach outlined above, various continuous measures of sum scores of neurologic signs, symptoms or nerve test scores, and scores of function of acts of daily living or of predetermined tasks or of disability. Irrespective of which approach is used, it is necessary to ensure good performance of evaluations with monitoring proficiency.

The first alterations observed in DPN patients are alteration in vibration perception threshold and reduction of ankle jerks [111]. The American Diabetes Association (ADA) recommended five simple examinations for DPN screening based on clinical signs: ankle reflex, pinprick sensation, temperature sensation, vibration perception thresholds, and pressure sensation [9]. NCS are the most precise tool for the detection of DN; along with symptoms and signs they give an accurate diagnosis. In 2010, the ADA suggested that NCSs should be considered as the gold standard for the diagnosis of DSPN [112].

One of the most employed items to detect the presence of DPN in clinical practice consists in the MNSI that includes the sum of scores varying from 0 to 1 for each abnormality revealed in foot appearance, Achilles reflexes presence, and vibratory threshold (VPT) by tuning fork (maximum score = 8). MNSI by using 2.5 score as cut-off may be considered a rapid, simple, reproducible, and reliable test for rapid ambulatory screening of PDN from the diabetologists [113]. The positive likelihood ratio of MNSI is 5.56 for those patients with ≥2.5 points of the composite score and raises up to 5.83 with >3 points [114].

Another tool for detection of DPN was validated in 2002, known as the Toronto Clinical Neuropathy Scoring System (TCSS), and evaluates the presence of symptomatic DPN (symptom, reflex, and sensory tests scores). It showed a significant negative correlation with sural nerve fiber density, and it was lower in those with better glycemic control. TCSS correlates well with the underlying structural damage in peripheral nerve as shown by the loss of myelinated nerve fibers [115]. In Table 1 we report a compilation of diagnostic methods for the diagnosis of DPN, with their respective sensitivity, specificity, positive predictive value (PPV), and negative predictive value (NPV).

### 2.5. Treatment

We focused on etiology-based treatment, leaving symptomatic treatment beyond our review. The basis of treatment for DPN and other microvascular complications of DM is glycemic control due to the basic mechanisms explained previously where excess of glucose is responsible for nerve damage and lack of regeneration. Once glucose levels normalize, nerve dysfunction can be stopped and the nerve fibers can improve [121].

#### 2.5.1. Metformin

The most worldwide used OA drug is still Metformin, a dimethylbiguanide, and the first line treatment for almost any kind of DM, but essential for type 2 DM. It has many advantages when compared to other OAs, such as lack of weight gain, low risk of hypoglycemia, and favorable effects on the lipid profile [122]. Beneficial effects of Metformin have also been observed in adipose tissue by reducing fatty acid oxidation, activation of adenosine monophosphate (AMP) kinase to increase glucose transporter (GLUT4) translocation, and reduction of gluconeogenesis in liver [123]. However, the reason why we included this particular OA is because it has the capacity to prevent mitochondrial permeability transition, reduces the risk of cell death, and has a mild inhibitory effect on complex I of the respiratory chain reaction of mitochondria, which traduce in cell protection from apoptosis induced by mitochondria-related toxicity of hyperglycemia [124]. Furthermore, another investigation of Metformin effects on mitochondria revealed that elevated glucose concentration leads to an oxidative stress that favors the mitochondrial permeability transition pore (PTP) opening and subsequent cell death in several endothelial cell types, and Metformin prevents this PTP opening-related cell death [125]. Kooy et al. studied the effect of Metformin added to insulin therapy in 390 patients with type 2 DM and macro- and microvascular complications; they discovered that Metformin reduced macrovascular complications at 4.3 years of follow-up, although no beneficial effects on DPN were observed [126]. Recently, Hasanvand et al. demonstrated that the activation of AMP kinase signaling pathway in diabetic neuropathy might be associated with the anti-inflammatory response, and Metformin reduced the levels of inflammatory cytokines in diabetic rats; it also improved motor nerve conduction velocities of the sciatic nerves [127].

Recent studies have addressed the importance of vitamin B12 deficiency among long-term users of Metformin as OA therapy. The prevalence varies depending on the cut-off point from 8.6 to 28.1%, and other known risk factors are DM duration ≥10 years, and concomitant use of proton pump inhibitors (PPI) or histamine H2 antagonists (H2A), but the clinical relevance of the deficiency is yet unclear [128, 129]. Ahmed et al. in 2016 published a cross-sectional study where vitamin B12 was measured in 121 type 2 DM patients and the association with DPN was evaluated. Forty-three (35.54%) patients had DPN and vitamin B12 deficiency was defined as levels <150 pmol/L. The prevalence of vitamin B12 deficiency was 28.1%; however, there was no association between vitamin B12 deficiency and DPN, and Metformin dose did not confer an increase risk on DPN presence [129]. Similar results were reported by other authors, with controversial results, but without strong evidence that vitamin B12 deficiency influences the presence or severity of peripheral neuropathy [130–132]. We recommend supplementation with vitamin B12 in those patients with long-term use of Metformin (≥10 years) or concomitant use of PPI/H2A, and evidence of clinical DPN. However, consider suspending supplementation if there is no evidence of improvement.

#### 2.5.2. Statins

The hydroxy methyl glutaryl-CoA reductases, also known as statins, have potent antioxidant properties evaluated in multiple clinical trials. Possible mechanisms reported are inhibition of NADPH oxidase, thus, reducing intracellular production of ROS and acting as free radical scavengers [133]. A subgroup of 136 patients with type 2 DM and diagnosis of DPN established by means of MNSI in the Fremantle Diabetes Study (FDS) were evaluated to observe the benefits of fibrates and statins. The results of a 5-year follow-up demonstrated that statins have a beneficial effect on the incidence of neuropathy, with a hazard ratio (HR) of 0.65 (CI 95% 0.46–0.93) [134]. Ezetimibe/Simvastatin and Rosuvastatin have been shown to reduce LPO after 16 weeks of treatment in type 2 DM patients with DPN, although no clinical outcomes were drastically changed compared to placebo [135]. However, a study performed in coronary artery disease patients where Ezetimibe/Simvastatin 10/20 mg was compared to Simvastatin 80 mg as monotherapy demonstrated that inflammation biomarkers (CRP, IL-6, monocyte chemoattractant protein-1, and soluble CD40) were unaltered after 6 weeks of treatment, probably explained by the theory that the target of statins resides on oxidative stress rather than inflammatory response [136]. A study performed in healthy male subjects treated with Simvastatin monotherapy showed no changes in oxidative stress to nucleic acids, LPO, and plasma antioxidants [137], probably due to the lack of free radicals increase, since Ezetimibe alone has been shown to reduce 8-isoprostanes and reactive oxygen metabolites levels only in hypercholesterolemic patients with high oxidative stress at baseline, but not in those with near-normal oxidative status [138]. Rosuvastatin 20 mg was assessed in 17 patients with type 2 DM and DPN, and a reduction of Neuropathic Symptoms Score (NSS) and NCS were observed after 12 weeks of treatment, along with a significant reduction in LPO but no changes in nerve growth factor beta [139]. Finally, both Atorvastatin and Rosuvastatin have proven an increase of total antioxidant capacity when given to type 2 DM patients with high low-density lipoprotein levels after 3 months of treatment, confirming the fact that statins increase the antioxidant status in patients with high levels of oxidative stress [140].

#### 2.5.3. Fenofibrate

A fibric acid derivative, Fenofibrate, is a peroxisome proliferator activated-receptor alpha (PPAR*α*) agonist recently approved for the management of diabetic retinopathy (DR). A subgroup of 1012 patients with DR aged 50–75 years who had type 2 diabetes in the Fenofibrate Intervention and Event Lowering in Diabetes (FIELD) study were assessed regarding the need for laser therapy, and it was found that Fenofibrate has an important effect reducing courses of laser treatment in patients with maculopathy or proliferative retinopathy [141]. The preliminary observational findings in the FDS indicate that fibrates have protective properties against neuropathy, with HR even lower than statins of 0.52 (CI 95% 0.27–0.98) [134]. An experimental study in* db/db* mice model of diabetic peripheral neuropathy published in 2014 demonstrated that Fenofibrate treatment ameliorated neural and endothelial damage by activating the PPAR*α*-adenosine monophosphate kinase- (AMPK-) PPAR*γ* coactivator- (PGC-) 1a-endothelial nitric oxide (eNOS) pathway [142]. Fenofibrate possesses anti-inflammatory, antioxidant, and anti-ischemic properties, and it could have beneficial effects on DPN, but large randomized clinical studies are needed to consider Fenofibrate an adequate treatment for DPN in type 2 DM patients [143].

#### 2.5.4. Ubiquinone (Coenzyme Q10)

Ubiquinone is a vitamin-like substance that contributes to adenosine triphosphate synthesis in mitochondrial electron transport chain. It is reduced to ubiquinol and redistributed into lipoproteins, possibly to protect them from oxidation [144]. In a study on diabetes-induced rat model where Coenzyme Q10 (CoQ10) was compared with *α* lipoic acid (ALA), the CoQ10 proved to stop the shift of actively contributing nerve fibers toward slower conduction velocities and tended to restore velocities of sciatic nerves toward those of the age-matched control group, whereas ALA did not produce statistically significant effects [145]. Clinical observation of endogenous CoQ10 has shown that, in type 2 DM patients, plasma and platelet MDA, as a marker of oxidative stress, were significantly higher, and the level of CoQ10, as antioxidant capacity, was significantly lower compared to controls, with a negative correlation between plasma CoQ10 and HbA1c [146]. When patients with type 2 DM and DPN were supplemented with 400 mg/day of CoQ10, NSS, Neuropathic Disability Score (NDS), and NCS were improved compared to placebo and associated with a reduction in LPO after 12 weeks of treatment [147]. Furthermore, an increase on total antioxidant capacity, anti-inflammatory effects, shown by reduction of CRP, and probably a protective effect on insulin resistance have been proven when supplementing with 200 mg/day of CoQ10 for 12 weeks, although, at this doses, no beneficial effects on clinical and nerve conduction were observed [148].

#### 2.5.5. Flavonoids

Polyphenols are useful nutraceuticals for type 2 DM patients. Some of their established effects are improving glycemic control, lipid profile, and insulin sensitivity. Furthermore, by regulating adipose metabolism, flavonoids can attenuate oxidative stress and modulate signaling pathways induced by ROS and inflammation [149]. In rat experimental models, an improvement of DPN was found with early intervention based on proanthocyanidins of the grape seed. They can also maintain the normal morphology of nerve tissue by reducing hyperglycemia and calcium overload in sciatic nerves [150]. Grape seed proanthocyanidins reduced low-density lipoproteins and enhanced nerve conduction velocity in Sprague-Dawley with induced type 2 DM [151]. One randomized, double-blinded, placebo-controlled clinical trial used QR-333, a topic drug with quercetin—a flavonoid contained in red wine—three times daily during 4 weeks for symptomatic DPN, and demonstrated a significant improvement in quality of life, neuropathic symptoms, with a good security profile. QR-333 reduced the severity of numbness, irritation, and pain when compared to placebo [152]. Another study evaluated the effect isoflavones-enriched bean sprouts on diabetic gastroparesis (autonomic neuropathy) in patients with type 2 DM with an improvement on gastric emptying compared to placebo [153]. Moreover, puerarin, an important isoflavonoid extracted from a Chinese herb* Puerariae radix, *has come into research attention, since a meta-analysis, where 22 clinical trials with 1664 subjects have investigated the efficacy of intravenous puerarin for DPN, concluded that combined with western medication it was more effective than conventional therapy for DPN in terms of total effective rate, nerve conduction velocity, and hemorheology index [154]. Naringenin is a flavone contained in citric fruits such as grapefruit and orange; it neutralizes oxidative stress and alterations in nerve growth factor in experimental DPN models [155, 156]. Hasanein and Fazeli demonstrated that long-term naringenin exposure has the capacity to exert significant analgesic and glucose lowering dose-dependent effects in a rat model with DPN [157]. In the same way, baicalein, a flavonoid originally isolated from the root* Scutellaria baicalensis, *has been used for many centuries in traditional herbal Chinese medicine for its antibacterial and antiviral properties [158]; it is a potent anti-inflammatory and antitumor agent, a free radical scavenger, and xanthine oxidase inhibitor, thus improving endothelial function and conferring cardiovascular protective actions against oxidative stress-induced cell injury. Its effects on DPN have not been proven yet, but because of the abovementioned properties it could exert beneficial effects on pathological nerve changes discussed here before. Finally, curcumin, a natural extract from* Curcuma longa* roots, exhibits antioxidant, antitumoral, and anti-inflammatory effects in experimental models [159]. It also promotes nerve regeneration and functional recovery after sciatic nerve injury in diabetic rats [160].

#### 2.5.6. Alpha-Lipoic Acid

Also known as thioctic acid, alpha-lipoic acid is a natural compound that acts as a cofactor for major complexes in mitochondrial enzymes. It contains two thiol groups capable of being oxidized or reduced. Its reduced form is named dihydrolipoic acid and its oxidized form as ALA. It can cross the hematoencephalic barrier and regenerate other antioxidants, such as vitamin C, vitamin E, and glutathione [161, 162]. The first series of studies addressing the capacity of ALA to exert beneficial effects on DPN were called ALADIN (Alpha-Lipoic Acid in Diabetic Neuropathy). In 1995, ALADIN I 328 non-insulin-dependent diabetic patients with symptomatic peripheral neuropathy were included and randomly allocated to one of three arms of treatment with 1200, 600, or 100 mg of intravenous ALA. The results were positive with significant reduction of symptoms score when compared to placebo after 19 days of treatment and a good security profile with the dose of 600 mg/day [163]. In 1999, ALADIN II was published with results after 24 months of treatment, initially with intravenous ALA for 5 days and then orally; even when a short number of patients were evaluated (27 in group 600 mg and 18 with 1200 mg/day), they evaluated the results with NCS and reported an improvement of sural sensory nerve conduction velocity and sural sensory nerve action potential for both arms. Tibiae motor nerve conduction velocity was only modified with 1200 mg/day [164]. Finally, in ALADIN III, 509 outpatients were tested for 6 months with 600 mg/day of ALA, with a reduction on neuropathic impairment score (NIS) but no changes in symptoms. The authors adjudged this unfavorable result possibly due to increasing intercenter variability in symptom scoring during the study [165]. ALA was included as the only etiologic treatment for DPN in the international guidelines, and some other studies have been conducted afterwards with favorable results even after 4 years of treatment [166–168].

#### 2.5.7. Aldose Reductase Inhibitors (ARI)

This group of drugs are intended to reduce the extent of polyol pathway deleterious events, by decreasing the accumulation of sorbitol in nerve fibers; however, as explained before, it is not the only pathway involved in the pathological findings of DPN [169, 170]. In experimental models a sorbitol dehydrogenase inhibitor did not have the expected results, and the effects on oxidative stress were counterproductive, by increasing MDA and 4-hydroxyalkenals, with reduced glutathione concentration [171]. Ranirestat is the most studied ARI in clinical trials. At first, in patients with mild to moderate DPN, great improvements in nerve conduction velocities above 1 m/s and vibration perception thresholds were observed, with promising results even in long-term evaluations [172, 173]; however, recent studies have been controversial, with no effect on efficacy endpoints and a mild improvement of less than 1.2 m/s in peroneal motor nerve conduction velocity with ranirestat [174] and no difference in clinical assessments compared to placebo [175].

#### 2.5.8. Miscellaneous

L-acetyl-carnitine (ALCAR) is a derivative from the amino acid carnitine that acts as a cofactor for lipid utilization as energy, mainly in the mitochondrial electron transport chain. ALCAR promotes regeneration of peripheral nerves in DPN in experimental studies [176].

## 3. Conclusions

There is still a lot of research to be done to fully understand the complex pathways in which hyperglycemia alters nerve function and even more regarding therapeutic approaches to reduce inflammatory, oxidative stress, and mitochondrial dysfunction, as part of the etiologic treatment for DPN. The lack of evidence with most of the treatments for DPN is associated probably with the selection of patients, since glucose control must be achieved in order to modify the overproduction of ROS/RNS; however, out of the clinical trials, the main problem of the diabetic population is the lack of prolonged glycemic control and specially the first years after the diagnosis of type 2 DM. If a prompt euglycemia is not accomplished, little or no effect of the abovementioned therapeutic approaches will continue to appear. The following clinical trials should be performed in early diagnosed DPN, and longer periods of time are needed to make further conclusions in terms of improvements. As an opinion of the authors in this review, for clinical trials, NCS should be assessed as an objective parameter for the evaluation of the therapies.

## Figures and Tables

**Table 1:** Diagnostic methods for DPN.

Author(s)	Diagnostic method	Sensitivity	Specificity	PPV	NPV
Single tests					
Dyck et al. [116]	Abnormal ankle reflex	60.3	90.5		
	Abnormal VPT	17.2	96.4		

Al-Geffari [117]	Abnormal ankle reflex	51.4	97.7	94.9	71.0
	10 g SW Monofilament	69.7	87.9	82.6	78.0
	128-Hz tuning fork	72.5	88.7	84.0	79.7

Composite scores					
Dyck et al. [116]	NIS (LL) + 7 tests	100	100		
	NIS (LL)	69	86.9		

Feldman et al. [118]	MNSI > 2	80	90	97	74

Moghtaderi et al. [114]	MNSI > 1.5	79	65	59	83
	MNSI > 2.0	65	83	71	79
	MNSI > 2.5	50	91	77	74
	MNSI > 3.0	35	94	80	70

Xiong et al. [119]	NSC	85.96	77.03	74.24	87.69
	NIS	59.65	98.65	97.14	76.04
	MNSI > 1.0	70.18	81.08	74.07	77.92
	MNSI > 1.5	57.89	97.30	94.29	75.00
	MNSI > 2.0	49.12	97.30	93.33	71.29
	MNSI > 2.5	36.84	98.65	95.45	66.97

Al-Geffari [117]	Combined tuning fork & SW Monofilament	89.5	84.9	92.8	89.5

Liu et al. [120]	TCSS	77.2	75.6		

Nerve conduction studies					
Dyck et al. [116]	≥1 nerve w/abnormal NCS	93.1	57.7		
	≥2 nerves w/abnormal NCS	81	91.2		
	≥3 nerves w/abnormal NCS	51.7	97.8		

DM, diabetes mellitus; IDDM, insulin-dependent diabetes mellitus; NIDDM, non-insulin-dependent diabetes mellitus; IGT, impaired glucose tolerance; MNSI, Michigan Neuropathy Screening.

Instrument; NCS, Nerve Conduction Study; NIS (LL), neuropathic impairment (disability) score of lower limbs; NSC, nerve symptomatic change score; SW, Semmes-Weinstein.
